# Wings as Part of the Sensory System in the Aphid Subfamily Eriosomatinae s. lat. (Insecta, Hemiptera)

**DOI:** 10.3390/insects16080828

**Published:** 2025-08-09

**Authors:** Barbara Franielczyk-Pietyra, Moshe Inbar, Paulina Hutyra, Łukasz Depa

**Affiliations:** 1 Institute of Biology, Biotechnology and Environmental Protection, Faculty of Natural Sciences, University of Silesia in Katowice, Bankowa 9, 40-007 Katowice, Poland; 2 Department of Evolutionary and Environmental Biology, University of Haifa, Haifa 3498838, Israel

**Keywords:** control proprioception, dispersal, flight, sensilla, sensing

## Abstract

Wings are a crucial evolutionary trait of insects and constitute an important part of their sensory system. Phloem-feeding aphids specialising in particular host plant species require an efficient sensory system and locomotive abilities to find an appropriate plant to complete the life cycle. Wings (in winged morphs) play a crucial role in this dispersal, but the sensory part of their structure is neglected. Here, we review campaniform sensilla, responsible for sensing the mechanical deformations of wings, in the aphid subfamily Eriosomatinae, where many species are host-alternating, which requires distant flights. Based on light and scanning electron microscopy, we illustrate sensilla and their arrangement on wings in 10 species. We also provide a broader sensory background by correlating their number with other important olfactory and campaniform sensilla of the antennae and legs. We find a strong relation of forewing sensilla with olfactory receptors on antennae, but their number is specific for particular morphs, depending on the required flight effort, led by the abundance of the host plant. Morphs migrating to less abundant host have 1.15 to 1.77 more campaniform sensilla per 1 mm of forewing length than morphs migrating to more abundant host. Aphids reveal a relatively high number of sensilla on the distal part of the forewings, showing their importance for effective sensing the deformation of wing.

## Simple Summary

Aphids are specialised feeders, requiring finding a proper host plant species to continue their life cycle. Special winged morphs, which were the subject of this study, perform flight in spring or autumn to continue their life cycle on different plants. Many sensory structures help them finding the required plant species, including sensory organs placed on wings, which help to detect the degree of deformation of wings during flight. Here, we used light microscopy and scanning electron microscopy to study these structures on forewings of aphids in the subfamily Eriosomatinae. It was revealed that aphids, which need to have longer flight due to rarity of the host plant they seek, have more specialised sensory structures, campaniform sensilla on their wings, than morphs searching for more abundant plants.

## 1. Introduction

The wings of insects are their crucial, innovative structures, having adaptations to many functions, including being part of the sensory system [1,2]. The sensory system of insects, including aphids, consists of a few types of sensilla, e.g., placoid, coeloconic, trichoid, and campaniform sensilla [3,4]. They serve as mechanoreceptors and chemoreceptors, crucial for receiving information on the aphid body position (e.g., feeding position), environmental disturbance (e.g., enemy approaching), finding proper host plant, and receiving signals from conspecifics, e.g., about colony density or presence of competing aphid species, or from mutualists, e.g., ants [5]. Therefore, the whole sensory system of aphids is quite complex, and sensilla are located on many parts of the body, primarily the olfactory antennal sensilla [6,7,8]. The whole aphid body is covered with trichoid sensilla, with additionally recognized sensilla on the legs, including trochanters, femora and tarsi, ultimate rostral segment, male genitalia, and wings [9].

Within this set of the sensory system, little attention has been given to wings, while winged aphid generations should find suitable primary or secondary hosts. Naturally, insect wings play a crucial locomotive role having proprioceptors, providing information on their position and movement [10]. So far, such receptors have been known mainly for bases of the forewings and hindwings in aphids as clearly visible clusters of small dots in light microscopy which have been revealed to be campaniform sensilla in scanning electron microscopy. Several further studies discovered the existence of such sensilla in other parts of the wings, usually along the main veins in the anterior part of the forewing [11,12]. Few studies provide information on the position and structure of these sensilla [11,13,14,15]. Montagano & Favret [12] for the first time presented detailed data on the distribution of campaniform sensilla on the wings of the aphid genus *Mindarus*. They showed a clear distribution pattern along the main veins of the wing without, however, an indication of the diagnostic importance of this pattern in the distinction of species.

Here, we provide a broader review of the wing sensilla of aphids within the subfamily Eriosomatinae. This is a relatively old taxonomic group of aphids, having obligatory sexual generation and predominating alternating between different host plant species. In spring, the alate females need to fly in search of the secondary host (in a broad sense these morphs are named *exules*—winged migrant females in spring and their offspring on the secondary host) or search for a primary host in autumn (winged sexuparous females) [16]. Some species may be anholocyclic—permanently parthenogenetic throughout the year, on a primary or secondary host. Another important feature of these aphids is a medium-to-dense wax cover, protecting them from excreted honeydew and living in open or closed galls on the primary host [17]. The winged morphs encounter various environmental conditions and have different adaptations. The spring migrant winged females (*fundatrigeniae*) need to find a secondary host where they can parthenogenetically bear the next generations. During summer, winged morphs (*exules*) proliferate to colonize new specimens of secondary host plants. In autumn (and in most Fordini in spring), a special generation of winged females (*sexuparae*) return to the primary host, predominately a tree species, where they bear sexual generation: dwarfish oviparous females and dwarfish males, both wingless, which copulate and female lays overwintering egg [18,19]. Each aphid species is more or less specialised to feed on a particular host plant species, which the migrant-winged female needs to find. It is a critical and difficult task (e.g., [20]).

Such broad polymorphism, feeding specialisations, and host alternation require efficient locomotive ability and vast sensory capabilities for migrant winged (alate) aphids. The aim of the study was to examine the distribution pattern of campaniform sensilla on their forewings. We correlate it with other sensilla of the aphid sensory system to find potential differences between morphs and species conducting different life modes and evaluate their potential taxonomic importance. The hypothesis was that due to increased difficulties with finding primary host plants in autumn, when weather conditions are more severe and flight time reduced by shorter day length, sexuparae will have more sensilla on forewings than alate fundatrigeniae, and such a difference may be constant across the studied species of Eriosomatinae s. lat.

## 2. Material and Methods

Materials for the light microscopy study were obtained from the entomological collection of the Institute of Biology, Biotechnology and Environmental Protection of the University of Silesia in Katowice (DZUS). The material for scanning electron microscope (SEM) studies was partly collected by the authors in 2023 and 2024 in Poland and partly in 2023 in Israel. The list of studied morphs with the number of specimens for a particular study method is presented in Table 1. The study comprised 139 specimens from 11 species and 10 genera based on 98 specimens for light microscopy and 41 specimens for scanning electron microscopy.

All studied specimens were winged females, either fundatrigeniae (born in spring by fundatrix on the primary host) or typical exuls—usually born in permanently parthenogenetic lines or born on secondary hosts, and sexuparae—autumn winged females bearing sexual generation, born on the secondary host and returning to the primary host (Table 1). For SEM studies, at least 2 individuals of each species were examined. The uneven number of studied specimens results from unavailability of properly mounted specimens of some species, with whole forewings evenly spread and preserved in a way enabling unambiguous counting of very pale and translucent sensilla.

Specimens with smaller amounts of the wax were cleaned with dish soap in an ultrasonic cleaner (Polsonic, Warsaw, Poland), with cleaning time dependent on specimen size while specimens more wax-covered were soaked overnight in 100% chloroform (Avantor Performance Materials Poland S.A., Gliwice, Poland). After cleaning, wings were dissected and dehydrated in a graded ethanol series of 80, 90, 96, and 100% for 10 min in each concentration, and then there were three 100% ethanol (Avantor Performance Materials Poland S.A., Gliwice, Poland) changes.

After that, samples were mounted on holders and sputter-coated with gold with the use of the turbomolecular pump coater (Quorum 150T ES plus—Quorum Technologies, Laughton, East Sussex, UK). Both sides of the wings were observed with the use of the SEM (Phenom XL, Phenom-World B.V., Eindhoven, The Netherlands) in the scanning microscopy laboratory of the Faculty of Natural Science, Institute of Biology, Biotechnology and Environmental Protection of the Silesian University in Katowice. Wings and legs of aphids were also analysed using a Nikon Eclipse E600 (Nikon, Amstelveen, The Netherlands) light microscope and photographed with a Nikon DS-Fi2 (Nikon, Amstelveen, The Netherlands) camera in the Laboratory of Insect Morphology and Anatomy, IBBEP Silesian University of Katowice. We followed the wing veins terminology after Wojciechowski [21] and Franielczyk-Pietyra & Wegierek [22]: costal vein (C), radial (R), media (M) and cubitus anterior vein (CuA), pterostigma (pt). Due to the fact that representatives of Eriosomatinae are known as well-covered with wax, despite cleaning, not all of the sensilla were well captured in the SEM images.

**Figure 1:**
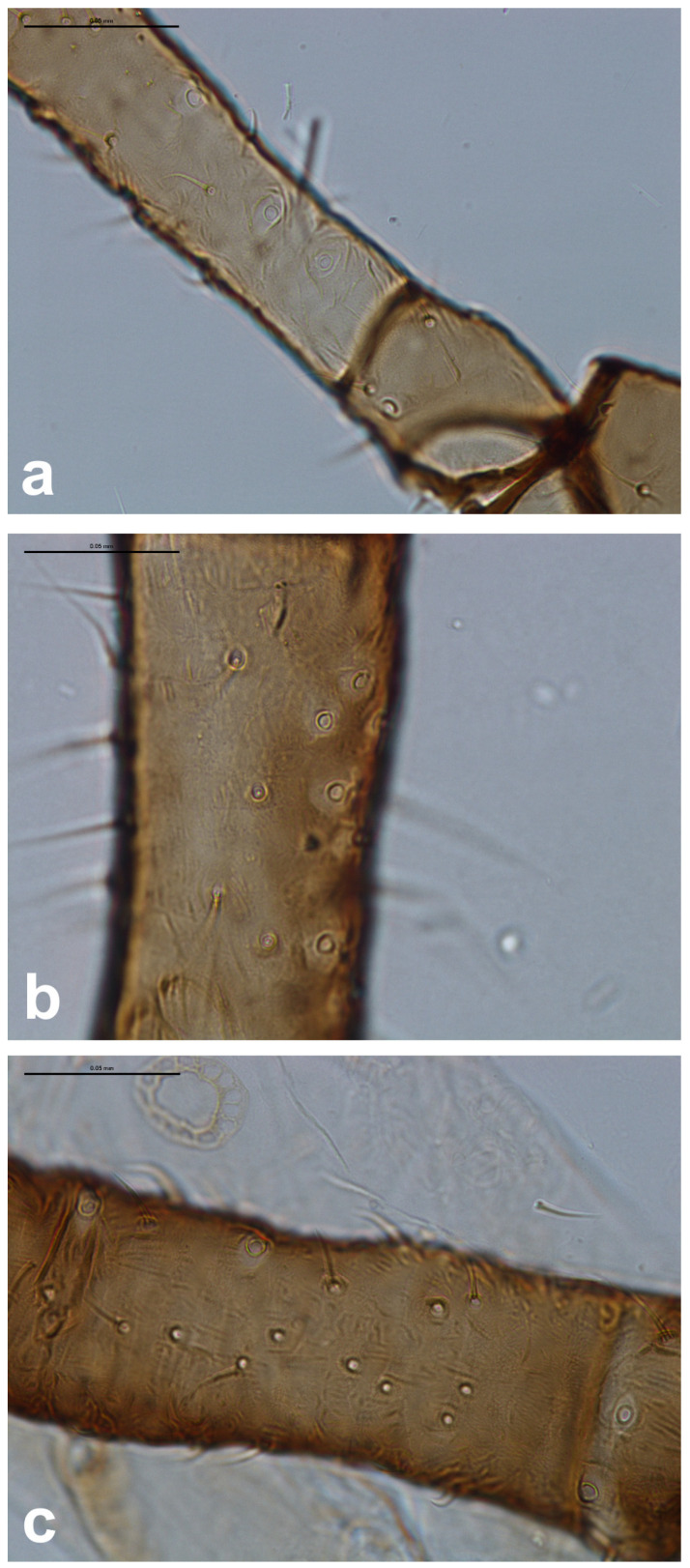
Campaniform sensilla located on the trochanters and bases of the femora: (**a**) *Eriosoma ulmi*, (**b**) *Prociphilus bumeliae*, (**c**) *Tetraneura ulmi*. Scale bar 0.05 mm.

The microscopic slides served to count campaniform sensilla on legs, forewings, and rhinaria on antennal segments. To study the relation of forewing campaniform sensilla to other sensilla of the sensory system of aphids, we chose rhinaria on antennal segments as they are prominent and crucial olfactory sensilla for aphids, serving as receptors of alarm pheromones and volatiles helping to find appropriate host plant species [8,23]. We additionally included campaniform sensilla located on trochanters and femora of aphids (Figure 1) (omitting tarsal ones as little informative) [24] as part of the proprioceptive part of the sensory system. We deliberately omitted any trichoid sensilla due to their vast placement throughout the body and difficulties with their recognition on wings due to, e.g., breaking out during mounting.

To check whether the combination of elements of the sensory system is specific for a particular species or morph, we performed a cluster analysis (the Minimum Variance method) using STATISTICA ver. 13.3 software (StatSoft). We analysed a set of sensilla as presented in Table 2. The morph of the studied specimens (alata exul (al.), fundatrigenia (fdg.) or sexupara (sxp.)) was also taken into account. The total number of analysed specimens was 98, although not all species were evenly represented (the number of specimens of particular species is listed in Table 1 for light microscopy). 

The relation between the forewing sensilla and other sensillar structures of the sensory system of aphids was evaluated via the correlation between the number of particular types of wing sensilla and other sensilla present on the aphid body, particularly the number of primary and secondary rhinaria on antennal segments III-VI and campaniform sensilla on trochanters and femora of all legs. The counted number of sensilla on these appendages was correlated with wing sensilla using Spearman’s correlation (Table 3). To avoid any uncertainties resulting from the unsatisfactory condition of the slides or errors while counting the sensilla, we applied the value *p* < 0.005 as the statistical significance threshold. We also measured the length of the wings to check the correlation between their length and the number of particular sensilla using the same method at *p* < 0.05. We counted the density of each type of campaniform sensilla per 1 mm of wing length (Table 4) by measuring the length of each studied wing of each specimen.

To compare the number of wing campaniform sensilla with certain biological traits of aphids (life cycle), we matched the number of sensilla of particular morphs with the theoretical difficulties in finding the proper host plant. We hypothesise that aphids that need to find rare host plants (the less abundant of required hosts) need to undertake more demanding flight and will have more sensilla on their wings.

We compared the abundance of particular host plants based on literature data, mainly geographical ranges and distribution of host plant species and genera [25,26,27,28,29], as it is difficult to receive and compare the detailed number of host plant specimens. Poaceae were always treated as the most abundant group of host plants. Special attention was given to a native range of species, as some of them have been massively cultivated also beyond their natural habitats (*Ribes* spp., *Picea abies*, *Abies alba*) [30,31]. Regarding the above difficulties, we only compared pairs of host plants for particular aphid species in relation to primary host vs. secondary host. Therefore, we only indicate whether the one host plant is more abundant (+) than the other (−). We compared it with the number of campaniform sensilla type III (as their number was the most variable) of the studied migrant alate females in reference to their life cycle. We also checked the differences between the numbers of the forewing campaniform sensilla (fcs) and fcs/wing length between two pairs: fundatrigenia vs. sexupara and morph flying along the gradient of the abundance of host plant vs. morph flying against the gradient. For these analyses, we used parametric Welch’s t-test and non-parametric Mann–Whitney U-test (Table 5) to enhance statistical power of the results if both tests give statistically significant results. Additionally, to have certainty that any correlations are related not only to the properties of the studied family, we used *Anoecia corni* of the family Anoeciinae as an outgroup. Anoeciinae are not related to Eriosomatinae but to Hormaphidinae, but their species have very similar life cycles and morphs, and they co-occur in the same area as the studied species of Eriosomatinae.

## 3. Results

The detailed results of measurements of particular sensory structures are presented in Table 2, with the addition of the performed life cycle and the form of the targeted host plant.

**Figure 2:**
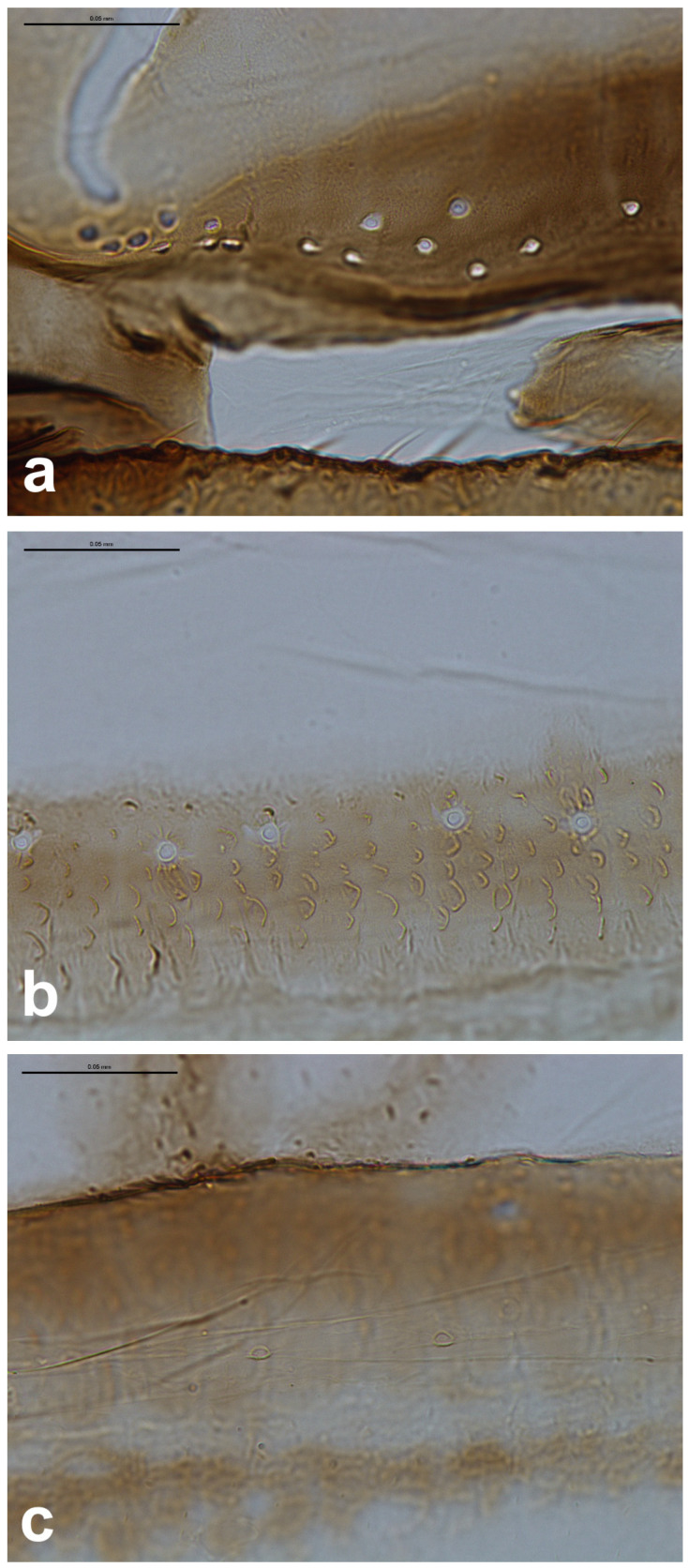
Campaniform sensilla located on the forewing of *Tetraneura ulmi* in light microscopy ((**a**)—fcs1, (**b**)—fcs2, (**c**)—fcs3). Scale bar 0.05 mm.

**Figure 3:**
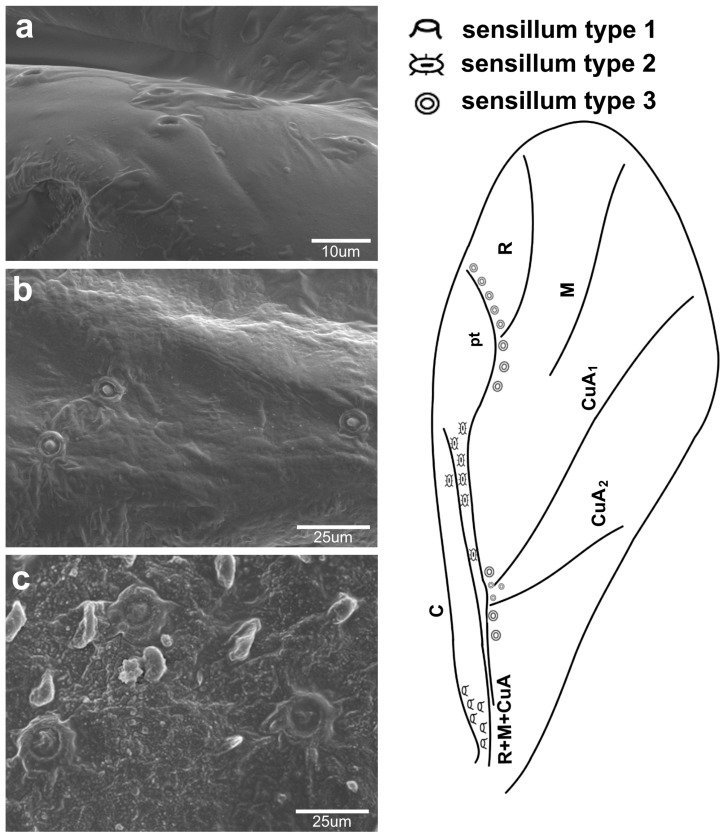
Campaniform sensilla located on the forewing of *Tetraneura ulmi* in scanning electron microscopy ((**a**)—fcs1, (**b**)—fcs2, (**c**)—fcs3) (**left**) and their arrangement on the wing (**right**).

**Figure 4:**
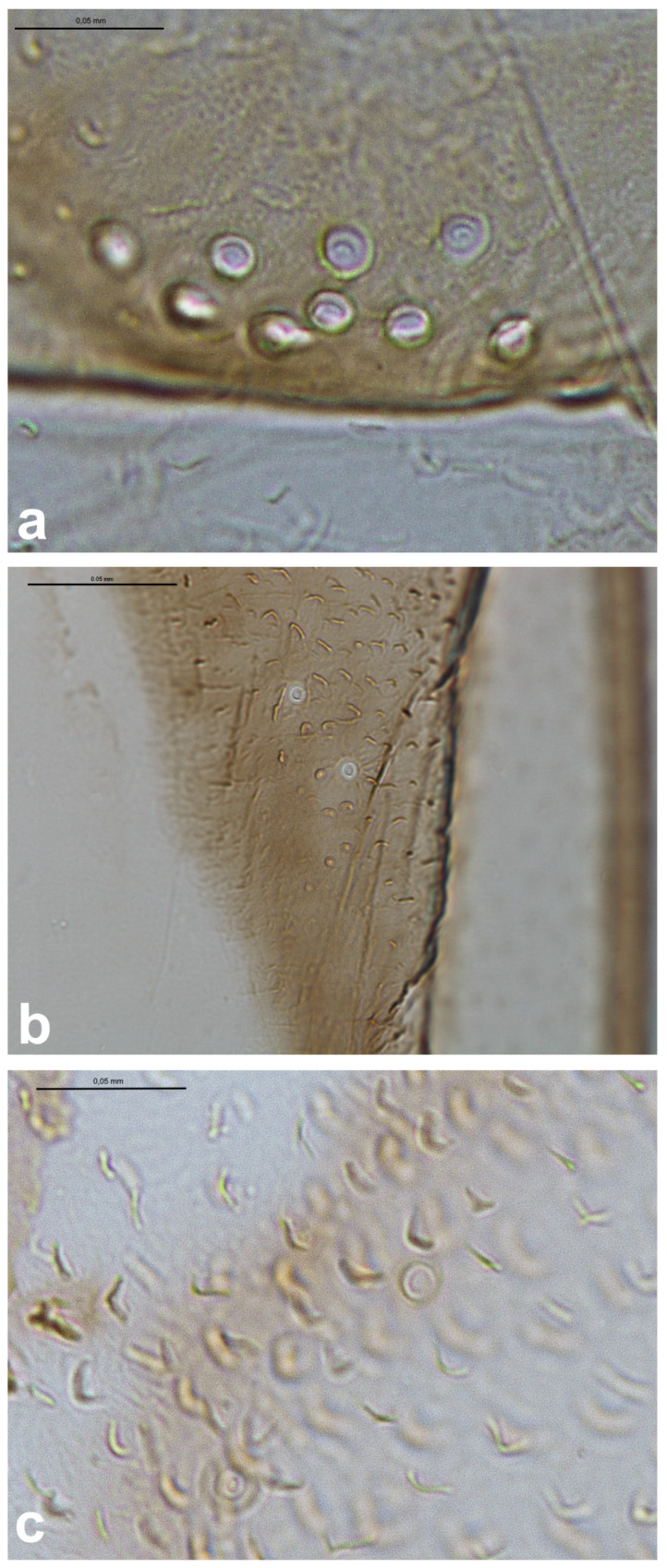
Campaniform sensilla located on the forewing of *Eriosoma ulmi* in light microscopy ((**a**)—fcs1, (**b**)—fcs2, (**c**)—fcs3). Scale bar 0.05 mm.

**Figure 5:**
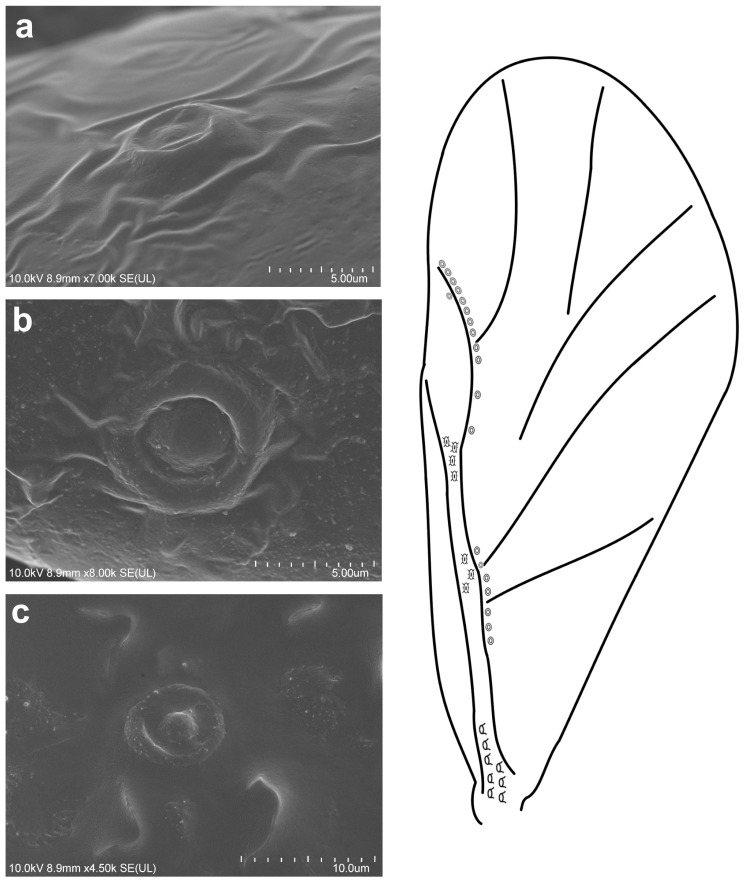
Campaniform sensilla located on the forewing of *Eriosoma ulmi* in scanning electron microscopy ((**a**)—fcs1, (**b**)—fcs2, (**c**)—fcs3) (**left**) and their arrangement on the wing (**right**).

**Figure 6:**
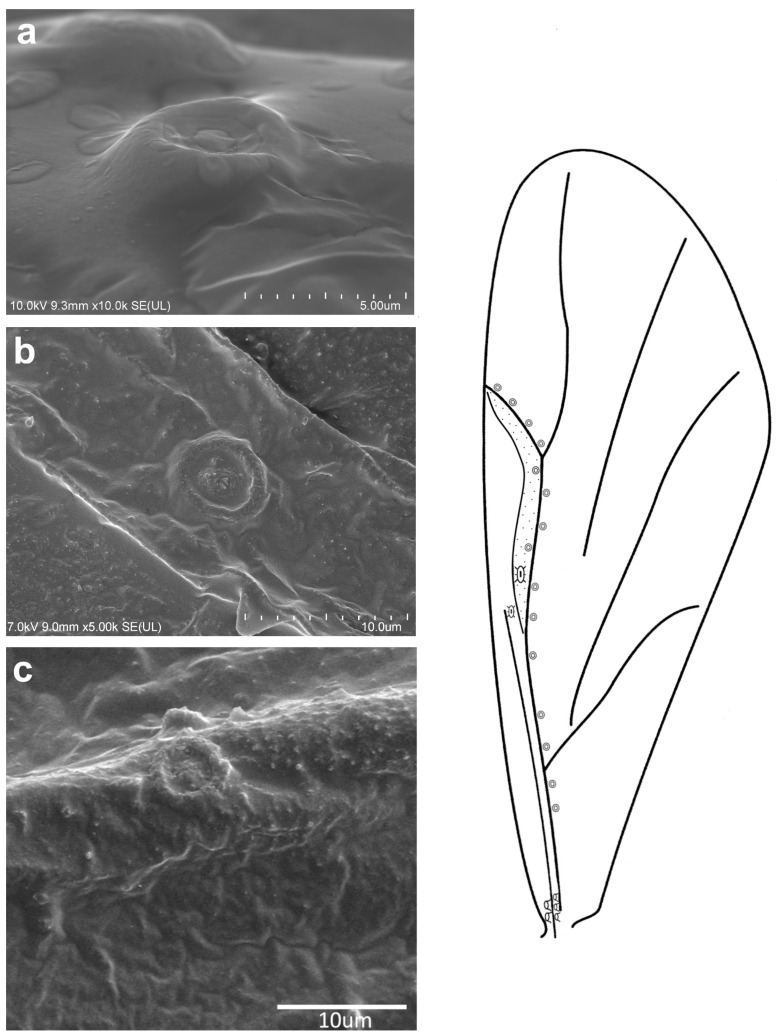
Campaniform sensilla located on the forewing of *Baizongia pistaciae* in scanning electron microscopy ((**a**)—fcs1, (**b**)—fcs2, (**c**)—fcs3) (**left**) and their arrangement on the wing (**right**).

**Figure 7:**
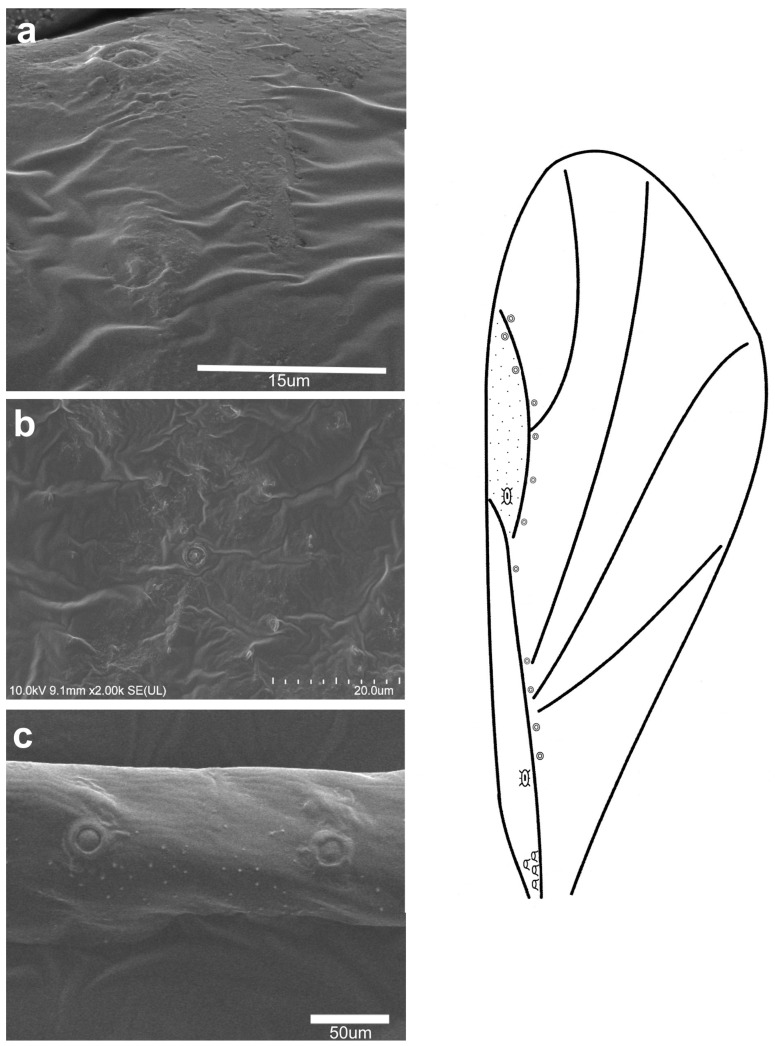
Campaniform sensilla located on the forewing of *Slavum wertheimae* in scanning electron microscopy ((**a**)—fcs1, (**b**)—fcs2, (**c**)—fcs3) (**left**) and their arrangement on the wing (**right**).

**Figure 8:**
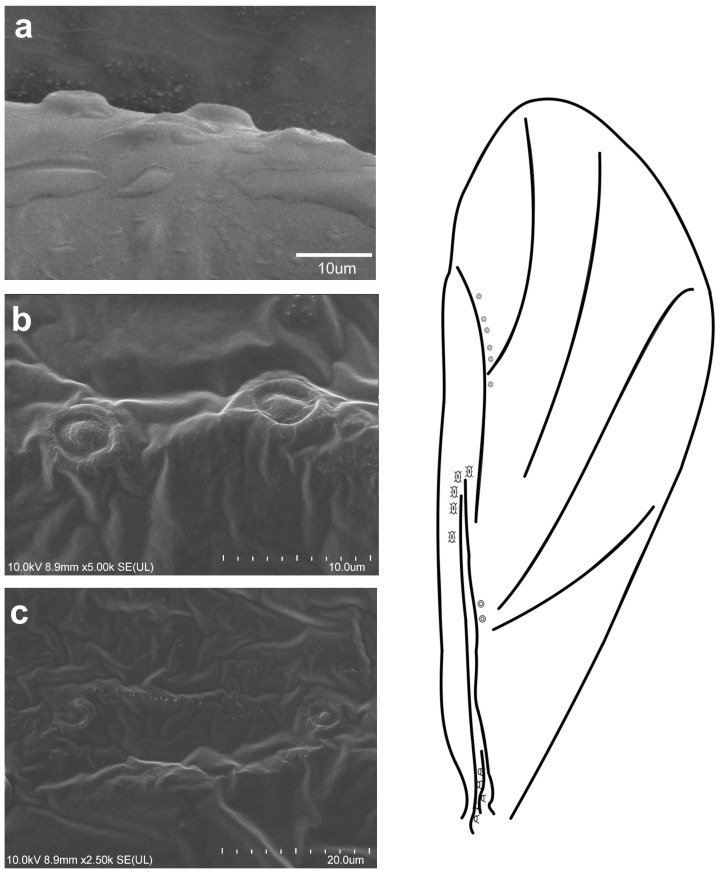
Campaniform sensilla located on forewing of *Mimeuria ulmiphila* in scanning electron microscopy ((**a**)—fcs1, (**b**)—fcs2, (**c**)—fcs3) (**left**) and their arrangement on the wing (**right**).

**Figure 9:**
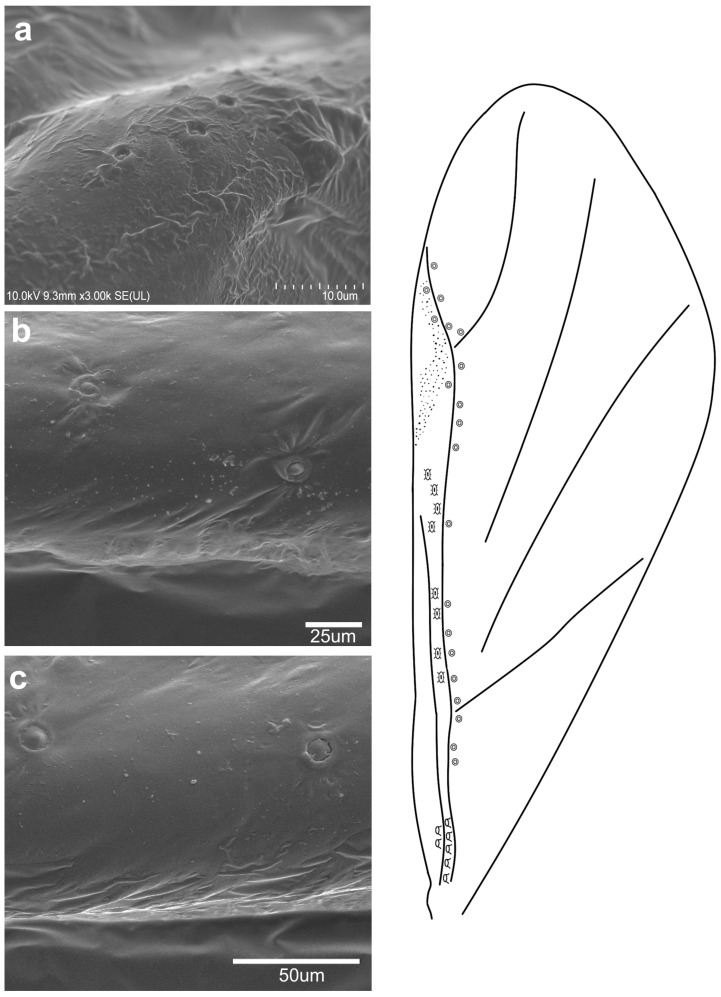
Campaniform sensilla located on forewing of *Prociphilus bumeliae* in scanning electron microscopy ((**a**)—fcs1, (**b**)—fcs2, (**c**)—fcs3) (**left**) and their arrangement on the wing (**right**).

The observed campaniform sensilla on the forewings of the studied aphids can be divided into three types according to their appearance in light microscopy and their position on the wing. The forewing campaniform sensilla type I (fcs1) comprises a sensilla on the dorsal side of the wing base, on the costal margin, usually distributed longitudinally over a short distance, heavily sclerotised and with sometimes weakly protuberant external margin (Figure 2a, Figure 3a, Figure 4a, Figure 5a, Figure 6a, Figure 7a, Figure 8a and Figure 9a).

**Figure 10:**
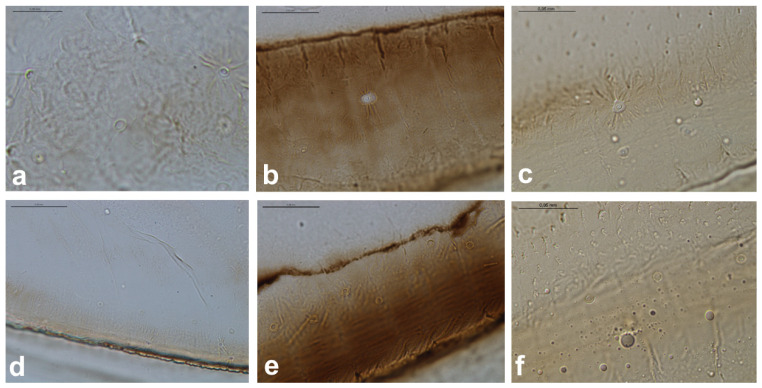
Campaniform sensilla located on the forewing of *Slavum wertheimae* (**left**), *Prociphilus bumeliae* (**middle**), and *Pachypappa tremulae* (**right**) in light microscopy ((**a**–**c**)—fcs2, (**d**–**f**)—fcs3). Scale bar 0.05 mm.

The forewing campaniform sensilla type II (fcs2) was located on the common stem composed of radial (R), media (M), and cubitus anterior (CuA) veins, R + M + CuA, on the dorsal side of the wing, from the base of the M vein to the base of the pterostigma (pt) (Figure 2b, Figure 3b, Figure 4b, Figure 5b, Figure 6b, Figure 7b, Figure 8b and Figure 9b). On this distance, they may be either clustered in a distal part near the pterostigma (Figure 3), more or less evenly scattered along this whole distance (Figure 5 and Figure 9), or occur singly near the base of the pterostigma (Figure 6, Figure 7 and Figure 8). In the light microscope, they looked like rings centred within the brighter oval spot in the darker vein sclerotisation, with longitudinal radial wrinkles (Figure 10a–c).

The forewing campaniform sensilla type III (fcs3) were located on the dorsal and ventral side of the wing, along the posterior margin of R + M + CuA to the tip of the pterostigma, distributed more or less evenly (Figure 2c, Figure 3c, Figure 4c, Figure 5c, Figure 6c, Figure 7c, Figure 8c and Figure 9c). In light microscopy, they were not clearly visible, and they had the appearance of flat, oval, or slightly elongated weakly sclerotised rings (Figure 10d–f).

All three types of sensilla were of approximately the same diameter in each species: fcs1: 4 μm (range 3–6 μm), fcs2: 6 μm (4–7 μm), fcs3: 6 μm (4–8 μm), but the fcs1 sensilla were slightly smaller than the remaining two types, which were also very similar in shape. As presented in Table 2, the separate morphs of a single species differ in the number of campaniform sensilla on their forewings. The mode of difference was not constant, e.g., in *Prociphilus bumeliae* and *Eriosoma ulmi*, winged fundatrigeniae had a higher number of fcs3 sensilla than sexuparae, while in *Tetraneura ulmi*, it was the opposite. In *E. ulmi* and *T. ulmi*, the difference between fcs3 sensilla and fcs1 and fcs2 in both morphs was also observable, and in *P. bumeliae*, it was observable in fcs1.

Spearman’s correlation coefficient indicated a solid correlation between the number of forewing sensilla of three types and the number of rhinaria on antennal segments III and IV, as well as the number of campaniform sensilla at the base of the second femur (Table 3, Figure 1). Additionally, (1) there was a significant correlation between the number of fcs2 sensilla and the number of rhinaria on antennal segment VI; (2) a correlation between the number of fcs1 and fcs3 sensilla and the number of sensilla on the base of the third femur; (3) strong correlation of fcs2 and fcs3 with wing length; and (4) a strong correlation between wing sensilla of all types to each other.

**Figure 11:**
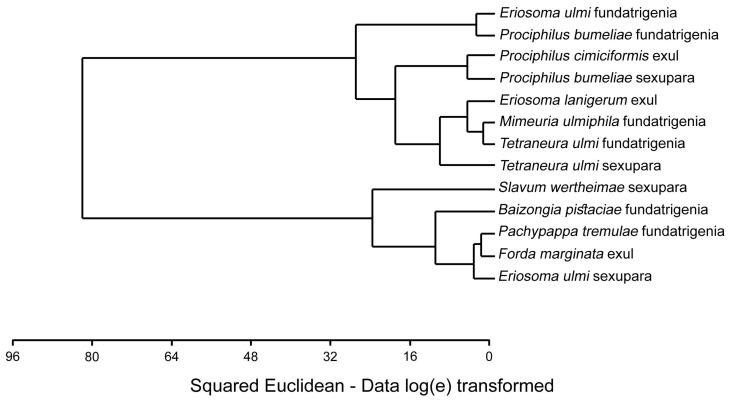
Cluster analysis of studied specimens regarding their morphs based on specimens examined in light microscopy (Ward method, Euclidean distances).

Cluster analysis did not follow any phylogenetic relationships between studied species, nor morphs, e.g., fundatrigeniae of *Prociphilus bumeliae* and *Eriosoma ulmi* were clustered together, while these species, according to some studies, may represent separate subfamilies (Figure 11). Similarly, fundatrigeniae of *Pachypappa tremulae* and sexupara of *Eriosoma ulmi* were clustered with various morphs of the tribe Fordini, where they do not belong. Although some clusters constitute taxonomic clades, e.g., *Slavum wertheimae* and *Baizongia pistaciae* (both Fordini), some species have been largely separated through morphs, e.g., *Eriosoma ulmi*. The results strongly indicate that the set of sensilla in the studied sensory systems is not directly typical of species but predominantly typical of particular morphs within the species, which may differ in the number of particular types of sensilla (Table 1). However, there was observed regularity between morphs, e.g., higher number of wing sensilla in either fundatrigenia or sexupara.

**Figure 12:**
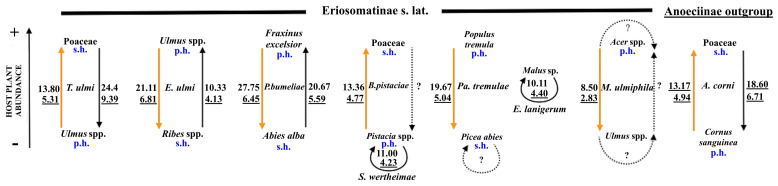
Relation between the number of forewing campaniform sensilla type 3 (numbers near arrows) and their density (underlined numbers near arrows) and the possible flight requirements of particular morphs in reference to the abundance of the host plant (left arrow indicates abundance: + increasing, − decreasing) (p.h—primary host, s.h.—secondary host; yellow arrow—flight of fundatrigenia, black arrow—flight of sexupara or exul (*E. lanigerum*); dotted lines—not studied but existing or possible migrations.

There were no statistical differences between the number of fcs of all types between fundatrigeniae and sexuparous morphs, as well as no statistically significant differences between fcs density of both studied morphs (Table 4 and Table 5). However, there were statistically significant differences in the number of all types of fcs between morphs flying in different gradients of host plant abundance (Table 5; Figure 12), with mean values “+” vs. “−”: fcs1—6.29 vs. 10.31, fcs2—5.16 vs. 7.53, and fcs3—12.91 vs. 22.33. In case of fcs density, such difference was recorded only in case of fcs3/wl (Table 5), where mean values of densities for “+” vs. “−” were the following: fcs1/wl—2.26 vs. 3.22, fcs2/wl—1.80 vs. 2.40, fcs3/wl—4.50 vs. 6.88.

## 4. Discussion

### 4.1. The Arrangement and Function of Fcs

The distribution and size of campaniform sensilla on the forewings of the studied Eriosomatinae are generally congruent with their arrangement in other insects [10], including aphids [11,12]. However, we provide the first detailed comparison between migrating morphs from several genera, as well as distinguish between these sensilla on wings. Montagano & Favret [12] do sort the fcs on the wings of *Mindarus* in reference to their position on the wing and the venation. Here, we did not follow this scheme because it was difficult to assign them to particular regions of the wing, as many sensilla of types 2 and 3 were quite evenly distributed along the wing, not clustered in certain regions. The only observed differences were between fcs2 and fcs3 in their appearance in light microscopy, as well as in their position. Fcs2 is always dorsally on the midline of R + M + CuA, surrounded by an areola of lighter chitinisation, while fcs3 is always posterior to R + M + CuA and pterostigma, dorsally and ventrally, weakly chitinised and almost translucent. Hence, the applied division in this subfamily was more evident and constant in all studied species than the division used by Montagano & Favret [12]. The distribution of fcs on the distal part of the wing (following the terminology by Aiello et al. [10]) is not typical for insects, although they are placed on the leading edge of the wing. Such an arrangement is suspected to inform of external perturbations, e.g., wings colliding with external objects, and provide better detection of wing deformation, especially by body rotation [10]. Considering the function of variously distributed sensilla on insect wings [32], it may be concluded that while the fcs1 location serves to sense the bending of the wing, the significant number of fcs3 on the distal part, especially near pterostigma, serves to sense shearing of the wing, most intense on its distal part. The quite significant number of sensilla on aphid forewings in comparison to other insects [10] shows their importance in aphid locomotory abilities by rapid neural transmissions through wings, reaching 2–3 ms [1]. This, together with olfactory and visual stimuli [33,34,35], affects aphids’ flight abilities.

### 4.2. Fcs as the Diagnostic Trait of Aphid Winged Morphs

It could be suspected that morphs within the species have a very similar pattern of wing campaniform sensilla. However, certain differences in the arrangement of antennal rhinaria observed within some species (e.g., number of rhinaria on antennal segments between spring and autumn morphs of *Prociphilus* spp.) also correlate with the number of campaniform sensilla on forewings (Table 3) (see Section 4.3) but the nature of mutual relation between these sensilla cannot be unambiguously resolved. As indicated by the cluster analysis (Figure 11), these arrangements do not follow phylogenetic relations between species (as shown by Montagano & Favret [12]) and do not follow morphs, which are often scattered in tree topology, not following any strict taxonomic or phylogenetic pattern. Observed differences between the morphs are not consistent, e.g., it is not always that alate fundatrigeniae have a higher or lower number of sensilla in comparison to winged sexuparae. The discrepancy with the positioning of various morphs of the same species on the cluster tree (Figure 11) indicates that there may be a difference in fcs arrangement also between alate fundatrigeniae and alate exuls from the secondary host. It must be further investigated whether differences in the number of fcs may differentiate between morphs in other groups of aphids. Observed inconsistency between fcs numbers and arrangement between morphs within species suggested that aphids’ winged morphs, despite being more ancestral in their structure than apterous morphs, may be functionally specialised forms [36], aiming at effective dispersal. The nature of this specialisation is not clear but our results suggest the different degree of development of sensillar structures in winged morphs having different flight target.

### 4.3. The Role of fcs3 in Aphid Migration

It seems that the number of wing campaniform sensilla type 3 may be associated with some challenges experienced during the flight, as already suggested in the literature [37], with studies showing that efficient fliers have more sensilla on forewings than poor fliers [38]. In each aphid species, the number of the fcs3 was higher in morphs that had to migrate to less abundant or more difficult to find plant species (Figure 12). In aphids that might continue their life cycle on the same plant species (sometimes on the same individual plant), the number of fcs3 was very low (Figure 12). From this fact, it may be concluded that mechanical deformation of wings during longer or more repeatable flights (due to repetitive punctures on various encountered plants) has adaptive value. It may be hypothesised that the higher number of these sensilla supports the flight to the host plant detected by antennal rhinaria by providing more information about deformations of wings. It is difficult to predict the efficiency of flight aimed at finding proper host plants in aphids. A study by Ward et al. [39] on *Rhopalosiphum padi* indicated that only 0.6% of autumn migrants find a host. Many migrating sexuparous females within Fordini land on the wrong host [40]. Therefore, it is suggested that the migration on a short distance, when proper hosts are near, is the most effective mode of dispersal [41]. However, the abundance of host plants may also be important, as shown by the results of our analysis. Aphids not involved in searching for a new host (e.g., *S. wertheimae, E. lanigerum, M. ulmiphila*—Figure 12) have a significantly lower number of fcs and the density of fcs3 per 1 mm of wing length (Table 5; Figure 12) than those undertaking long flights in search of crucial host plant species (e.g., *E. ulmi*, *T. ulmi*. *P. bumeliae*). In some cases, e.g., *E. ulmi*, both host plants (*Ulmus* spp. and *Ribes* spp.) occupy the same habitat, moist-to-wet forests, Alno-Ulmion (e.g., [42,43]), so the probability of finding each plant is relatively high on short-distance flight. In the case of *Prociphilus* (having the highest number of fcs3 among all studied species, occurring in spring alate, Table 2; Figure 12), abundance and range of *Fraxinus excelsior* and *Abies alba* are distinct, although overlapping, with *F. excelsior* abundant throughout Europe [44]; however, *A. alba* is mostly limited to montane areas [45]. Therefore, finding *A. alba* for spring migrant is more demanding than finding *F. excelsior* for autumn migrant, though it is not followed by olfactory sensilla, as spring alata has fewer antennal rhinaria than autumn migrant. The difficulty with finding host plants also seems to affect other studied species. For instance, *T. ulmi* spring alate has fewer antennal rhinaria, as finding grass roots seems to be unproblematic, but spring alate of *E. ulmi* has many more antennal rhinaria than autumn migrant, as finding small shrubs of *Ribes* spp. in spring seems to pose more difficulties than finding tree trunks of *Ulmus* spp. in autumn (Table 2; Figure 12). The pattern of fcs3 in *A. corni*, a species from a different subfamily but with a similar life cycle, living in similar climatic conditions follows the one recorded in Eriosomatinae, indicating that this may be a more general pattern in host-alternating aphids.

Taking into account the observed correlations between the studied parts of the sensory system, it seems that they are mutually interconnected and provide aphids with a set of stimuli crucial for the proper functioning of winged aphids as migrating morphs. The correlation between the number of campaniform sensilla on antennae and legs may be another indication of the evolutionary history of wings as exites of ancestral legs of insects [46]. Special relation links olfactory receptors on rhinaria and forewing campaniform sensilla of type 3. Because both these types of sensilla provide aphids with distinct information and yet are strongly correlated, it may indicate their congregative effect enhancing the aphid directional dispersal.

## 5. Conclusions

Research confirmed the important role of the wings as a sensory organ in aphids, showing a variable number of campaniform sensilla on the forewings of aphid species within a single subfamily. However, the number of forewing campaniform sensilla of type 3 is associated with the migratory task. Morphs needing to seek less abundant plants may have more forewing campaniform sensilla of type 3 on their wings.

## Figures and Tables

**Table 1:** List of species, their morphs, and a number of specimens of aphids studied in both microscopic techniques (X—missing specimens).

Subfamily	Tribe	Species	Studied Morphs and the Number of Studied Specimens		
				Light Microscopy	Scanning Electron Microscopy
Eriosomatinae	Eriosomatini	*Tetraneura ulmi* (Linnaeus, 1758)	spring alate—fundatrigenia/exul	10	2
			autumn alate—sexupara	8	X
		*Eriosoma ulmi* (Linnaeus, 1758)	spring alate—fundatrigenia	9	5
			autumn alate—sexupara	7	X
		*Eriosoma lanigerum* (Hausmann, 1802)	spring alate—exul	9	6
	Fordini	*Baizongia pistaciae* (Linnaeus, 1767)	spring alate—exul	9	9
		*Slavum wertheimae* (Hille Ris Lambers)	autumn alate—sexupara	9	6
		*Forda marginata* (Koch, 1857)	summer alate—exul	2	X
		*Paracletus cimiciformis* (von Heyden, 1837)	summer alate—exul	1	X
	Pemphigini	*Mimeuria ulmiphila* (Del Guercio, 1917)	spring alate—fundatrigenia	2	2
		*Prociphilus bumeliae* (Schrank, 1801)	spring alate -fundatrigenia	4	5
			autumn alate—sexupara	6	6
		*Pachypappa tremulae* (Linnaeus, 1761)	spring alate—fundatrigenia	3	X
Anoeciinae—outgroup		*Anoecia corni* (Fabricius, 1775)	spring alate—fundatrigenia	12	X
			autumn alate—sexupara	10	X

**Table 2:** Mean number of particular sensilla on antennal segments, legs, and forewings of studied species of aphids based on light microscopy (abbreviations: fdg.—alate fundatrigenia, sxp.—alate sexuparous female, al.—alate female, possibly exul; N—number of studied specimens; ant—number of antennal segment; troch—trochanter, fem—femur; H—holocyclic species, A—anholocyclic on primary (1) or secondary (2) host; D—diecious species, M—monoecious).

		Rhinaria on Antennal Segments				Sensilla on Trochanters and Femora						Forewing Campaniform Sensilla (fcs)			Wing Length			
	N	ant III	ant IV	ant V	ant VI	troch 1	fem 1	troch 2	fem 2	troch 3	fem 3	Type 1	Type 2	Type 3		Life Cycle	Host Alternation	Flight Destination
*T. ulmi* fdg.	10	12.50	3.10	6.40	1.50	4.10	4.10	4.00	3.50	4.00	3.40	6.30	5.40	13.80	2.60	H(A2)	D	grass roots
*T. ulmi* sxp.	8	15.80	3.20	9.10	1.00	4.00	4.20	3.90	3.60	4.11	4.78	10.60	7.40	24.40	2.61			tree trunk
*E. ulmi* fdg.	9	39.67	6.22	1.00	1.00	4.00	6.56	4.00	4.56	4.00	6.67	8.11	6.78	21.11	3.10	H	D	shrub roots
*E. ulmi* sxp.	7	13.71	2.29	1.00	1.00	4.00	2.29	3.86	2.43	3.86	2.86	7.57	4.86	10.33	2.50			tree trunk
*P. bumeliae* fdg.	4	18.50	5.50	1.00	1.00	4.25	5.00	4.25	5.50	4.50	5.50	11.25	6.50	27.75	4.30	H(A2)	D	tree roots
*P. bumeliae* sxp.	6	26.33	12.50	12.00	8.60	4.00	3.83	4.00	5.17	4.33	4.17	7.00	7.83	20.67	3.70			tree trunk
*M. ulmiphila* fdg.	2	13.00	7.00	5.50	1.00	4.00	4.00	4.00	2.50	4.00	3.00	5.00	6.00	8.50	3.00	H(A1.2)	D	tree branches
*Pa. tremulae* fdg.	3	8.00	1.33	1.00	1.00	4.00	3.33	4.00	3.67	4.00	5.00	6.67	7.67	19.67	3.90	H(A2)	D	tree branches
*E. lanigerum* al.	9	22.56	4.67	4.89	1.22	4.00	5.11	4.00	4.11	4.00	5.89	7.11	3.89	10.11	2.30	A	M	tree branches
*S. wertheimae* sxp.	9	1.00	1.00	1.00	1.00	4.00	3.75	4.00	1.89	4.00	1.00	4.22	1.89	11.00	2.60	H	M	tree gall
*B. pistaciae* al.	9	6.27	3.18	2.27	1.00	3.91	4.91	4.00	3.55	4.00	4.82	5.82	2.09	13.36	2.80	H(A2)	D	tree trunk
*A. corni* fdg.	12											7.67	5.00	13.17	2.63	H	D	grass roots
*A. corni* sxp.	10											10.50	8.70	18.60	2.99			shrub leaves

**Table 3:** Spearman’s correlation coefficients between numbers of sensilla on particular structures (ant—antennal segment; fem—femur, troch—trochanter; pairs with no significant values omitted; values statistically significant (at *p* < 0.005 for number of sensilla, *p* < 0.05 for wing length) marked bold; ρ > 0.6 underlined).

	Rhinaria on Antennal Segments				Sensilla on Trochanters and Femora				Forewing Campaniform Sensilla		
	ant III	ant IV	ant V	ant VI	fem 1	fem 2	troch 3	fem 3	Type 1	Type 2	Type 3
ant IV	** 0.84 **										
ant V	0.28	** 0.46 **									
ant VI	0.31	** 0.36 **	** 0.60 **								
fem 1	** 0.42 **	** 0.41 **	−0.03	0.00							
troch 2	0.00	0.10	0.09	0.00	0.05						
fem 2	** 0.60 **	** 0.61 **	0.27	0.30	** 0.36 **						
fem 3	** 0.64 **	** 0.51 **	0.02	0.01	** 0.65 **	** 0.66 **	0.08				
type 1	** 0.55 **	** 0.35 **	0.04	0.12	0.23	** 0.45 **	0.14	** 0.48 **			
type 2	** 0.65 **	** 0.55 **	0.29	** 0.41 **	0.05	** 0.47 **	0.21	0.28	** 0.54 **		
type 3	** 0.44 **	** 0.42 **	−0.01	0.25	0.26	** 0.56 **	** 0.33 **	** 0.44 **	** 0.51 **	** 0.59 **	
wing length	0.18	0.40	−0.10	−0.19	0.06	0.60	** 0.68 **	0.34	0.20	** 0.64 **	** 0.65 **

**Table 4:** Values of the density of fcs per 1 mm of wing length (wl), and the direction of morph flight along the gradient of the abundance of the host plant (+) and against it (−).

	fcs1/wl	fcs2/wl	fcs3/wl	Flight
*T. ulmi* fdg.	2.42	2.08	5.31	+
*T. ulmi* sxp.	5.75	3.07	9.39	−
*E. ulmi* fdg.	2.62	2.19	6.81	−
*E. ulmi* sxp.	3.03	1.94	4.13	+
*P. bumeliae* fdg.	2.62	1.51	6.45	−
*P. bumeliae* sxp.	1.89	2.12	5.59	+
*M. ulmiphila* fdg.	1.67	2.00	2.83	+
*Pa. tremulae* fdg.	1.71	1.97	5.04	−
*E. lanigerum* al.	3.09	1.69	4.40	+
*S. wertheimae* sxp.	1.62	0.73	4.23	+
*B. pistaciae* al.	2.08	0.75	4.77	+
*A. corni* fdg.	2.91	1.91	4.94	+
*A. corni* sxp.	3.41	3.29	6.71	−

**Table 5:** Results of statistical analysis of the number of fcs and fcs/wl ratios for pairs of morphs: fundatrigeniae (fdg, n = 6) vs. sexuparae (sxp, n = 5) and morphs flying along the gradient of host plant abundance (+, n = 6) and against (−, n = 5), (n—number of studied cases of morphs); values of statistical importance marked bold (at *p* < 0.05).

		Welch’s *T*-Test	Mann-Whitney U-Test	
		*p*	Z	*p*
fcs1	fdg. vs. sxp.	0.529	−0.263	0.792
	+ vs. −	**0.046**	**−2.166**	**0.030**
fcs1/wl	fdg. vs. sxp.	0.338	−0.823	0.410
	+ vs. −	0.244	−1.189	0.234
fcs2	fdg. vs. sxp.	0.982	−0.437	0.662
	+ vs. −	**0.031**	**−2.166**	**0.030**
fcs2/wl	fdg. vs. sxp.	0.570	−0.618	0.537
	+ vs. −	0.171	−1.158	0.247
fcs3	fdg. vs. sxp.	0.938	0.087	0.931
	+ vs. −	**0.004**	**−2.380**	**0.017**
fcs3/wl	fdg. vs. sxp.	0.512	−0.263	0.792
	+ vs. −	**0.024**	**−2.380**	**0.017**

## Data Availability

Materials used for this study are stored in the IBBEP University of Silesia, Katowice (contact the corresponding author). The datasets used and analysed during the current study are available from the corresponding author on reasonable request.

## References

[1] Pratt B., Deora T., Mohren T., Daniel T. (2017). Neural evidence supports a dual sensory-motor role for insect wings. Proc. R. Soc. B.

[2] Salcedo M.K., Socha J.J. (2020). Circulation in insect wings. ICB.

[3] Snodgrass R.E. (1926). The morphology of insect sense organs and the sensory nervous system, *Smithson*. Misc. Collect..

[4] Snodgrass R.E. (1935). Principles of Insect Morphology.

[5] Yang L.L., Wang B., Shen J., Wang G.R. (2023). Comparative morphology and plant volatile responses of antennal sensilla in *Cinara cedri* (Hemiptera: Lachninae), *Eriosoma lanigerum* (Hemiptera: Eriosomatinae), and *Therioaphis trifolii* (Hemiptera: Calaphidinae). Front. Cell. Neurosci..

[6] Shambaugh G.F., Frazier J.L., Castell A.E.M., Coons L.B. (1978). Antennal sensilla of seventeen aphid species (Homoptera: Aphidinae). Int. J. Insect Morphol. Embryol..

[7] Park K.C., Hardie J. (1998). An improved aphid electroantennogram. J. Insect Physiol..

[8] Park K.C., Hardie J. (2002). Functional specialisation and polyphenism in aphid olfactory sensilla. J. Insect Physiol..

[9] Kanturski M., Świątek P., Trela J., Borowiak-Sobkowiak B., Wieczorek K. (2020). Micromorphology of the model species pea aphid *Acyrthosiphon pisum* (Hemiptera, Aphididae) with special emphasis on the sensilla structure. Eur. Zool. J..

[10] Aiello B.R., Stanchak K.E., Weber A.I., Deora T., Sponberg S., Brunton B.W. (2021). Spatial distribution of campaniform sensilla mechanosensors on wings: Form, function, and phylogeny. Curr. Opin. Insect Sci..

[11] Watase A. (1962). Studies of the sense organ on the wing of aphids. J. Agric. Sci..

[12] Montagano L., Favret C. (2016). The Distribution of Campaniform Sensilla on the Appendages of *Mindarus* Species (Hemiptera: Aphididae). Entomol. News..

[13] Dickerson B.H., Aldworth Z.N., Daniel T.L. (2014). Control of moth flight posture is mediated by wing mechanosensory feedback. J. Exp. Biol..

[14] Kanturski M., Ali Akbar S., Favret C. (2017). Morphology and sensilla of the enigmatic Bhutan pine aphid *Pseudessigella brachychaeta* Hille Ris Lambers (Hemiptera: Aphididae)—A SEM study. Zool. Anz..

[15] Kanturski M., Lee Y. (2024). *Miyalachnus*—A New Lachninae Aphid Genus from Japan (Insecta, Hemiptera, Aphididae). Insects.

[16] Heie O. (1980). The Aphidoidea (Hemiptera) of Fennoscandia and Denmark—I. Fauna Entomologica Scandinavica.

[17] Inbar M., Schultz J.C. (2001). Once again, insects worked it out first. Nature.

[18] Wool D. (2004). Galling aphids: Specialization, biological complexity, and variation. Annu. Rev. Entomol..

[19] Purkart A., Morawski M., Masłowski A., Depa Ł. (2019). Ant-mediated anholocyclic overwintering of *Prociphilus fraxini* (Hemiptera: Aphididae) in Central Europe. Entomol. Fenn..

[20] Wool D., Manheim O., Inbar M. (1997). Return Flight of Sexuparae of Galling Aphids to Their Primary Host Trees: Implications for Differential Herbivory and Gall (Aphidoidea: Pemphigidae: Fordinae) Abundance. Ann. Entomol. Soc. Am..

[21] Wojciechowski W. (1992). Studies on the Systematic System of Aphids (Homoptera, Aphidinea). Pr. Nauk. Uniw. Śląskiego W Katowicach.

[22] Franielczyk-Pietyra B., Wegierek P. (2017). The forewing of the *Aphis fabae* (Scopoli 1763) (Hemiptera, Sternorrhyncha): A morphological and histological study. Zoomorphology.

[23] Anderson M., Bromley A.K., Minks A.K., Harrewijn P. (1987). Sensory System. Aphids. Their Biology, Natural Enemies and Control.

[24] Watase A. (1962). Studies of the sense organ on the legs of aphids. J. Agric. Sci..

[25] Svennig J.C., Skov F. (2004). Limited filling of the potential range in European tree species. Ecol. Lett..

[26] Kozhoridze G., Orlovsky N., Odlovsky L., Blumberg D.G., Golan-Goldhirsh A. (2015). Geographic distribution and migration pathways of *Pistacia*—Present, past and future. Ecography.

[27] Beck P., Caudullo G., Tinner W., de Rigo D., San-Miguel-Ayanz J., de Rigo D., Caudullo G., Houston Durrant T., Mauri A. (2016). Fraxinus excelsior in Europe: Distribution, habitat, usage and threats. European Atlas of Forest Tree Species.

[28] Caudullo G., de Rigo D., San-Miguel-Ayanz J., de Rigo D., Caudullo G., Houston Durrant T., Mauri A. (2016). Populus tremula in Europe: Distribution, habitat, usage and threats. European Atlas of Forest Tree Species.

[29] Baeten L., Bruelheide H., van der Plas F., Kambach S., Ratcliffe S., Jucker T., Allan E., Ampoorter E., Barbaro L., Bastias C.C. (2019). Identifying the tree species compositions that maximize ecosystem functioning in European forests. J. Appl. Ecol..

[30] Caudullo G., Tinner W., de Rigo D., San-Miguel-Ayanz J., de Rigo D., Caudullo G., Houston Durrant T., Mauri A. (2016). Picea abies in Europe: Distribution, habitat, usage and threats. European Atlas of Forest Tree Species.

[31] Mauri A., de Rigo D., Caudullo G., San-Miguel-Ayanz J., de Rigo D., Caudullo G., Houston Durrant T., Mauri A. (2016). Abies alba in Europe: Distribution, habitat, usage and threats. European Atlas of Forest Tree Species.

[32] Hinson B.T., Morgansen K.A. (2015). Gyroscopic sensing in the wings of the hawkmoth *Manduca sexta*: The role of sensor location and directional sensitivity. Bioinspir. Biomim..

[33] Taylor G.K., Krapp H.G. (2007). Sensory systems and flight stability: What do insects measure and why?. Adv. Insect Phys..

[34] Webster B. (2012). The role of olfaction in aphid host location. Physiol. Entomol..

[35] Schröder M.L., Glinwood R., Ignell R., Krüger K. (2017). The role of visual and olfactory plant cues in aphid behaviour and the development of non-persistent virus management strategies. Arthropod Plant Interact..

[36] Powell G., Tosh C.R., Hardie J. (2006). Host plant selection by aphids: Behavioral, evolutionary, and applied perspectives. Annu. Rev. Entomol..

[37] Gorelkin V.S., Karelin Y.A., Svidersky V.L., Gribakin F.G., Wiese K., Popov A.V. (1990). Proprioceptive control of flight in insects. Sensory Systems and Communication in Arthropods.

[38] Gnatzy W., Grünert U., Bender M. (1987). Campaniform sensilla of *Calliphora vicina* (Insecta, Diptera) I. Topography. Zoomorphology.

[39] Ward S.A., Leather S.R., Pickup J. (2001). Mortality during dispersal and the cost of host-specificity in parasites: How many aphids find hosts?. J. Anim. Ecol..

[40] Wool D., Manheim O., Burstein M., Levi T. (1994). Dynamics of re-migration of sexuparae to their primary hosts in the gall forming Fordinae (Aphidoidea: Pemphigidae). Eur. J. Entomol..

[41] Loxdale H.D., Loxdale H.D., Hardie J., Halbert S., Foottit R., Kidd N.A.C., Carter C.I. (1993). The relative importance of short- and long-range movement of flying aphids. Biol. Rev..

[42] Biurrun I., Campos J.A., Mijangos I.G., Herrera M., Loidi J. (2016). Floodplain forests of the Iberian Peninsula: Vegetation classification and climate features. Appl. Veg. Sci..

[43] Matisone I., Kaupe D., Matisons R., Klavina D. (2023). Understory changes in mixed elm stands in response to canopy dieback in Latvia. Balt. For..

[44] Marigo G., Peltier J.-P., Girel J., Pautou G. (2000). Success in the demographic expansion of *Fraxinus excelsior* L. *Trees*
**2000**, *15*, 1–13. Trees.

[45] Gazol A., Camarero J.J., Gutiérrez E., Popa I., Andreu-Hayles L., Motta R., Nola P., Ribas M., Sangüesa-Barreda G., Urbinati C. (2015). Distinct effects of climate warming on populations of silver fir (*Abies alba*) across Europe. J. Biogeogr..

[46] Dickinson M.H., Hannaford S., Palka J. (1997). The evolution of insect wings and their sensory apparatus. Brain Behav. Evol..

